# Multiple synchronous primary malignant neoplasms of renal, ureter and urinary bladder: a case report

**DOI:** 10.3389/fonc.2025.1576764

**Published:** 2025-07-24

**Authors:** Bincheng Huang, Wenhui Yin, Mohammed Abdulkarem Al-Qaisi, Haifu Tian, Mengmeng Zhao, Jin Zhang, Chaodan Deng, Na Li, Rui He, Guangyong Li

**Affiliations:** ^1^ Urology Department of General Hospital, Ningxia Medical University, Yinchuan, China; ^2^ Key Laboratory of Fertility Preservation and Maintenance of Ministry of Education, Ningxia Medical University, Yinchuan, China

**Keywords:** synchronous, MPMN, urinary, verrucous carcinoma, kidney

## Abstract

Synchronous multiple primary malignant neoplasms (MPMNs) involving distinct histopathological entities within the upper urinary tract represent a rare clinical phenomenon. We present a novel case of a 57-year-old male presenting with asymptomatic gross hematuria, subsequently diagnosed with three concurrent malignancies: papillary renal cell carcinoma(T1bN0M0), verrucous carcinoma of the renal pelvis (T1N0M0), and low-grade invasive urothelial carcinoma of the ureter (T2N0M0). Comprehensive imaging evaluation revealed a 5.7*5.2cm renal mass and 12cm ureteral tumor extending into the bladder. Following radical nephroureterectomy, histopathological confirmation of three distinct primary malignancies was achieved. Postoperative surveillance identified metastatic progression to adrenal, retroperitoneal, and peritoneal regions within four months, with subsequent development of bladder urothelial carcinoma. The unusual coexistence of three histologically distinct upper urinary tract malignancies, particularly the rare verrucous carcinoma variant, provides novel insights into tumor biology and clinical management paradigms for genitourinary MPMNs.

## Introduction

MPMNs encompass the concurrent or consecutive emergence of two or more distinct pathological tissue types within the same organ or tissue, or across various organs or tissues within the same individual (1).Recent advancements in tumor diagnosis and treatment technology have led to a perceived rise in clinical reports documenting MPMNs (2).This case is worth mentioning because the clustering of four primary malignancies (synchronous and metachronous) is of rare occurrence in a single patient and to our knowledge, this is the first report of the combination of three synchronous primary cancers of the upper urinary tract appearing in the same patient.

In this case report, we highlight a unique patient scenario characterized by the presence of three distinct histological types of malignant neoplasms within the upper urinary tract. Specifically, the patient was diagnosed with renal papillary renal cell carcinoma, verrucous carcinoma of the renal pelvis, and low-grade uroepithelial carcinoma in the ureter.

## Case report

**Figure 1:**
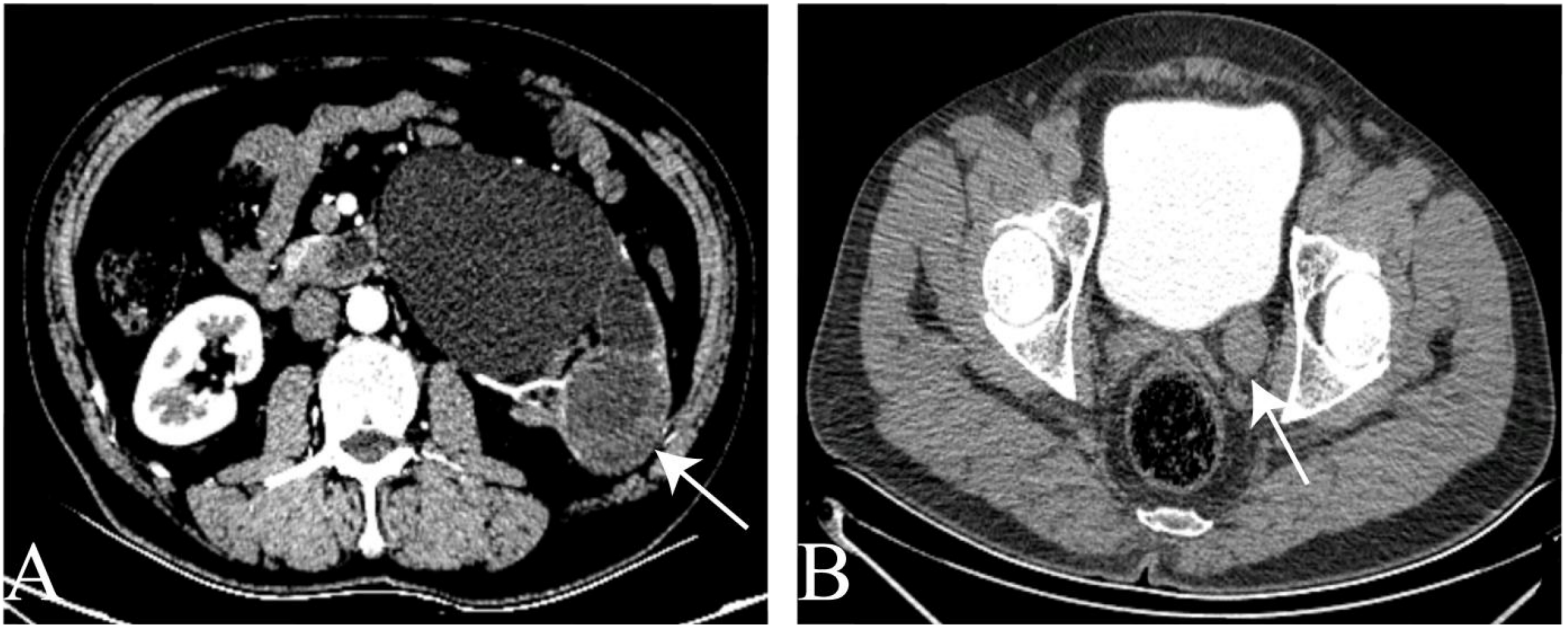
The CTU image. **(A)** space occupying lesion in the lower pole of the left kidney. **(B)** The tumor of the left ureter.

A 57-year-old male presented to the urology department on July 16, 2022, with asymptomatic gross hematuria. Physical examination revealed kidney percussion tenderness, while the rest of the clinical assessment was unremarkable. The patient’s medical history included hypertension, with no smoking or diabetes mellitus. Subsequent abdominal ultrasonography identified a 6.8x5.7 cm lesion in the left kidney. Computed tomography reconstruction of the urinary tract (CTU) revealed a 5.7*5.2 cm cystic, solid lesion located in the lower pole of the left kidney. Additionally, a 12 cm long mass was observed in the lower left ureter, extending into the bladder, with contrast enhancement (**Figure 1**).

**Figure 2:**
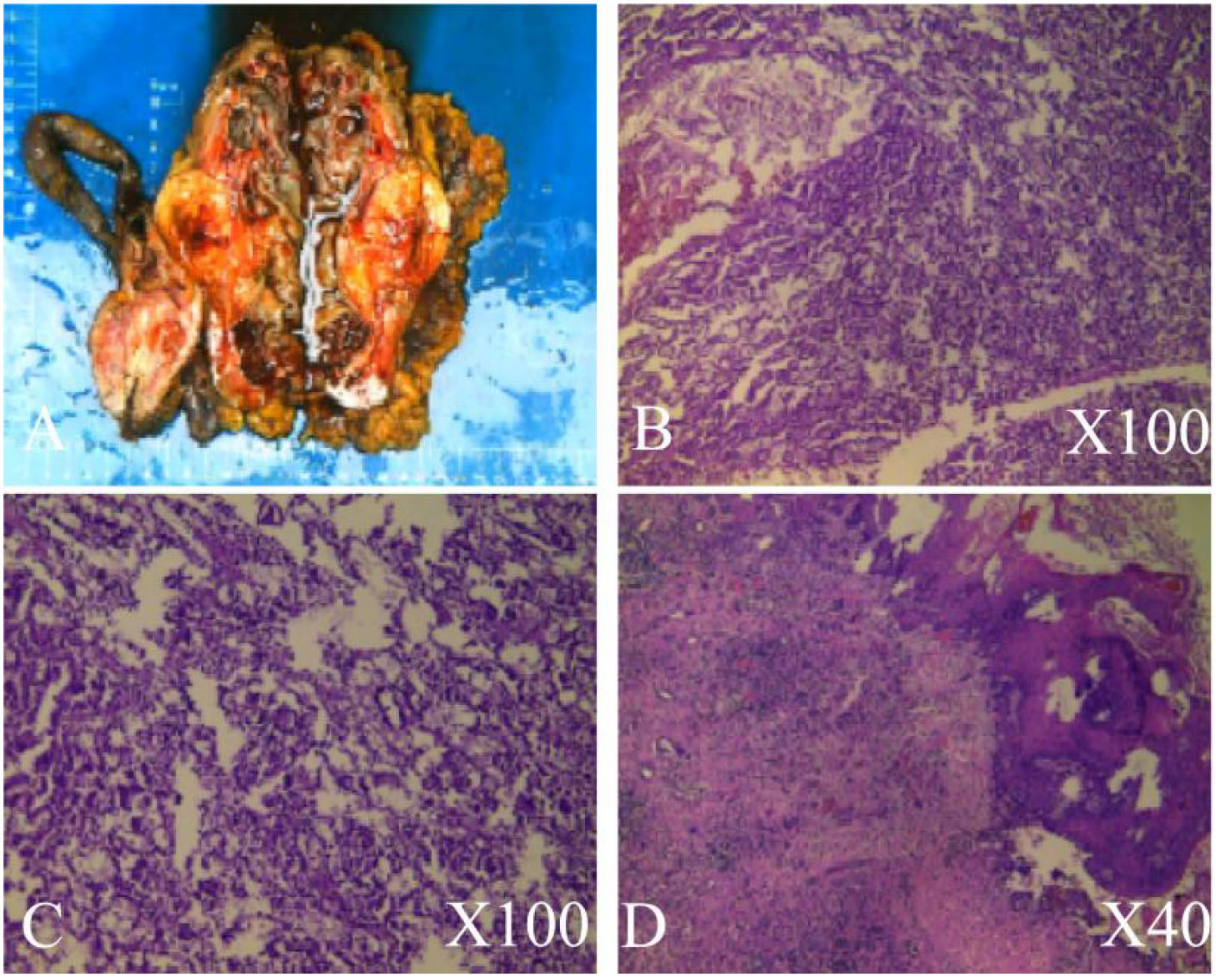
Histopathological examination. **(A)** Excised kidney tissue, kidney tumor, ureter, and perinephric fat. **(B)** Pathological features of papillary renal cell carcinoma. **(C)** Pathological features of verrucous carcinoma of the renal pelvis. **(D)** Pathological features of low-grade invasive uroepithelial carcinoma in the ureter.

The CT scan showed no tumors apart from those in the ureter and kidney, and no metastases were detected. Following comprehensive auxiliary examinations, surgical intervention was conducted under general anesthesia. On July 20, 2022, a left laparoscopic nephroureterectomy was performed, resulting in the complete resection of the left kidney and left ureter, including the tumor. The surgical specimen measured 18*7*5cm (**Figure 2A**), and it was subsequently sent for histopathological examination. The histologic findings revealed three distinct diagnoses:1. Papillary renal cell carcinoma (type I) (**Figure 2B**) staged as T1bN0M0.2. Verrucous carcinoma of the renal pelvis (**Figure 2C**) with cancerous tissue invading the submucosa, staged as T1N0M0.3. Low-grade invasive uroepithelial carcinoma of the ureter (**Figure 2D**) with invasion into the smooth muscle of the ureter, staged as T2N0M0.

**Figure 3:**
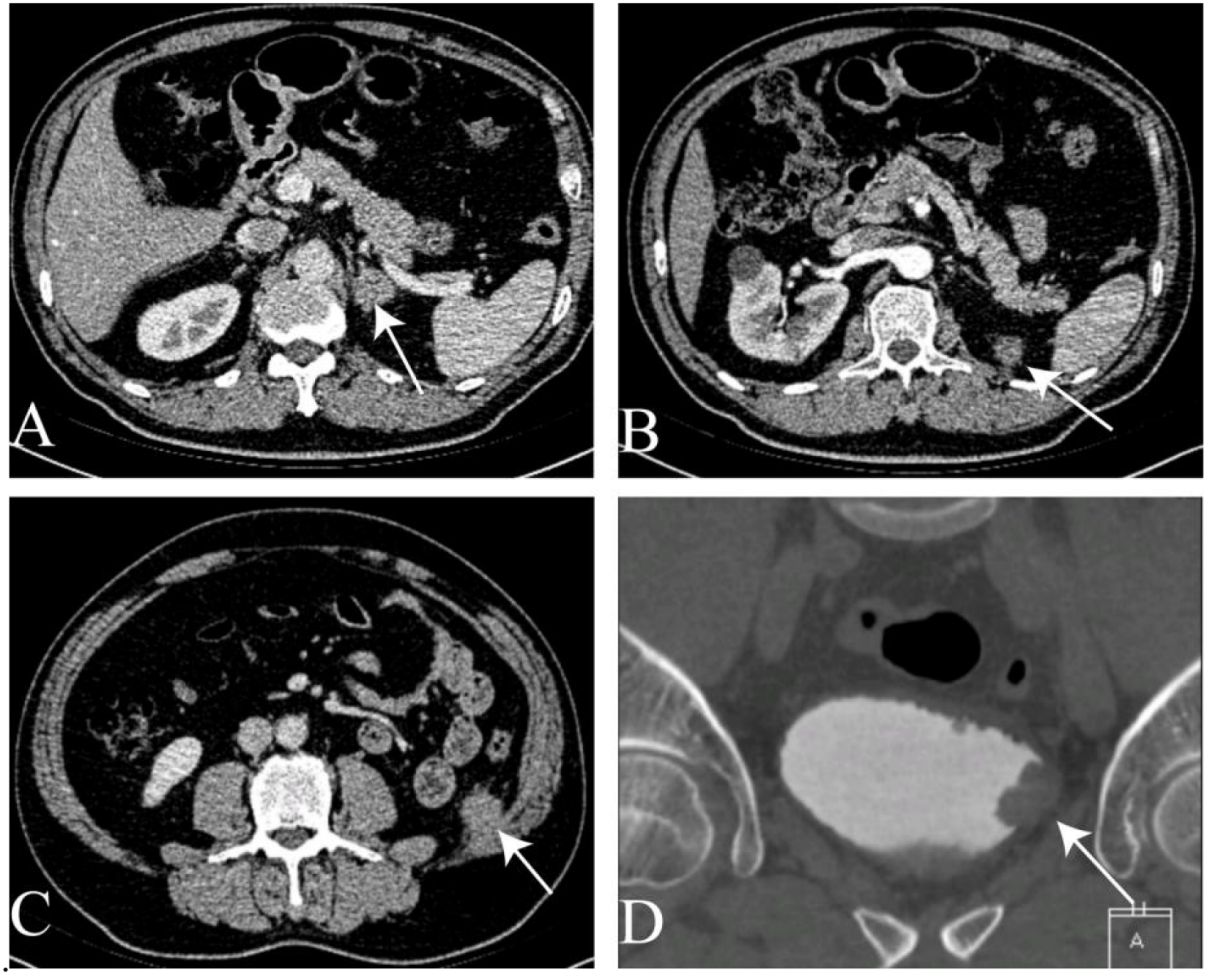
Computed tomography of the abdomen. **(A)** Left adrenal mass. **(B)** Mass in the left retroperitoneum and peritoneum. **(C)** Mass in the abdominal wall. **(D)** Mass in the bladder.

Computed tomography of the entire abdomen was conducted every 3-6 months post-surgery. Four months later, abdominal CT imaging on November 2, 2022, revealed left adrenal metastasis (**Figure 3A**). Metastases were also observed in the left retroperitoneal area, peritoneum (**Figure 3B**), and abdominal wall (**Figure 3C**). One month thereafter, CTU on November 24, 2022, identified a suspicious lesion on the left bladder wall (**Figure 3D**).

**Figure 4:**
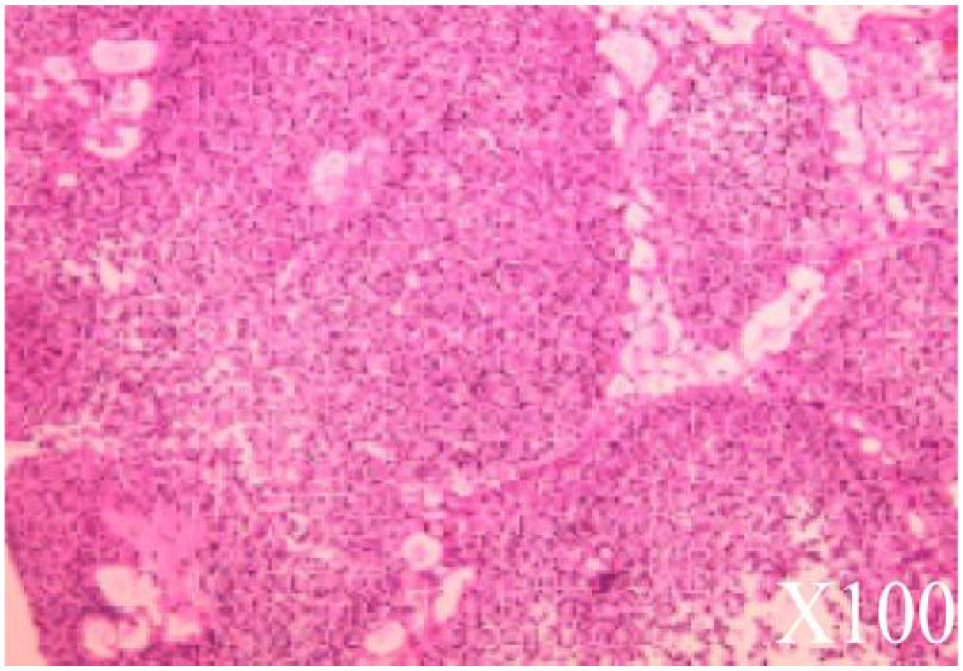
Pathological representation of urothelial carcinoma of the bladder.

The patient underwent Transurethral Resection of Bladder Tumour (TURBT) on December 1, 2022, for suspected recurrent bladder tumors. Multiple cauliflower-like tumors were observed scattered throughout the bladder during surgery. The bladder tumor was completely excised and submitted for histopathological examination. The histological analysis revealed low-grade uroepithelial carcinoma, staged as cT2bN1M1 (**Figure 4**).

Two weeks post-surgery, the patient commenced chemotherapy at the standard dosage: gemcitabine (1.8g) and cisplatin (150mg) administered every three weeks. Regular follow-up visits were scheduled, including hematological assessments, ultrasonographic examinations, and abdominal CT scans. Clinical chemistry parameters, renal and liver function tests, urinalysis, as well as vital signs such as height and weight, were diligently monitored. Enhanced abdominal CT imaging conducted on March 13, 2023, revealed a soft tissue shadow measuring 6.8 x 5.4 x 3.4 cm with uneven density enhancement in the left kidney area. Additionally, a soft tissue mass approximately 8.6 x 6.1 cm was observed in the left abdominal wall, accompanied by enlarged lymph nodes around the abdominal aorta region. By April 2023, the patient reported experiencing pain and hematuria during regular follow-up evaluations.

## Discussion

The occurrence of multiple primary malignant tumors in the urinary tract is a rare phenomenon in clinical practice, presenting significant technical challenges in treatment. The incidence of such occurrences in the urinary system is reported to be approximately 6.31% in international studies (3).MPMNs originate from various organs and systems. The location of predilection varies according to the distribution of tumor occurrence in each region and is usually related to the patient’s environment (4),family history (5),genetic (6)and tobacco smoking (7) (7).However, it’s noteworthy that the patient in this case had no history of smoking, no significant past medical history, and no family history of carcinomas. Particularly, there was no family history of malignancies with young onset among any family members.

MPMNs were initially described by Bilroth in 1889 (8). In 1932, Warren and Gates proposed three criteria for the diagnosis of a second primary cancer: i) each tumor must present a definite clinical and histological picture of malignancy; ii) each tumor must be histologically distinct; and iii) the probability that one was a metastatic lesion from the other must be excluded (9).

Synchronous malignancies are defined as those occurring within 6 months of the diagnosis of a previous malignant neoplasm, while metachronous malignancies are those that occur more than 6 months apart (10), heterochronous malignancies comprise the majority of cases. In this instance, the patient initially presented with asymptomatic gross hematuria accompanied by kidney percussion pain. At the first visit, three concurrent upper urinary tract cancers were identified. Four months later, bladder tumors and metastases developed. Thorough histopathological examination and adequate sectioning facilitated the successful diagnosis of three distinct primary malignancies within the upper urinary tract. Consequently, the patient is classified as having MPMNs.

The genitourinary system is the preferred site for MPMNs, with bladder and prostate cancers being the most commonly observed (11).However, the proportion of renal and ureteral cancers combined with MPMNs is even lower. It has been reported in the literature that renal cancer accounted for 2% of the first MPMN cancers and 2.4% of the second primary cancers (12).In this case, the occurrence of renal verrucous carcinoma is particularly rare. Verrucous carcinoma, a distinctive variant of squamous cell carcinoma (SCC), is uncommon but notable. It was first described by Lauren Ackerman in 1948 (13).Verrucous carcinoma can manifest at various sites, including the upper aerodigestive tract, skin, bladder, and genitalia (14).The renal tumor in this case exhibits typical features of verrucous carcinoma. Verrucous carcinoma refers to a distinct histologic appearance characterized by well-differentiated squamous cell carcinoma with localized infiltration and minimal propensity for metastasis to regional lymph nodes or distant sites, as observed in our tumor. Although bladder metastases developed post-surgery, they did not exhibit verrucous carcinoma characteristics. Additional histologic features include extensive keratinization with exophytic or papillomatous extension into the stroma and a lack of significant cellular anaplasia (15). Verrucous carcinoma is recognized as a warty variant of squamous cell carcinoma, featuring predominantly exophytic growth of well-differentiated keratinizing epithelium with minimal atypia. It typically exhibits locally destructive pushing margins at its interface with underlying connective tissue and does not invade the lamina propria. The tumor cells demonstrate little atypia, although faint invasion foci may be observed in verrucous carcinomas (16).

There is limited literature available nationally and internationally regarding verrucous carcinoma. Additionally, upper urothelial carcinoma is considered a relatively rare condition, accounting for approximately 5–10% of all urothelial cancers (17). In Western countries, the incidence of upper urothelial carcinoma is low, estimated at approximately 1–2 cases per 100,000 individuals. It is predominantly diagnosed in older male patients (18). In this case, the patient presented with a combination of three urological tumors, representing simultaneous MPMNs. Instances of three or more primary upper urinary malignancies are rare, with few reports documented in the literature.

An early diagnosis of MPMNs holds the potential to facilitate timely administration of anticancer treatments, including surgery, chemoradiotherapy, and immunotherapy, thereby improving overall survival and progression-free survival rates. However, there are currently no established guidelines for the treatment of MPMNs, and management typically relies on the primary cancer that appears at different times. Nevertheless, in cases of multiple primary malignancies of the urinary tract, surgery is often prioritized in treatment approaches. For elderly patients with multiple primary malignancies of the urinary tract who are in poor health, non-operative treatment may be considered if surgery is deemed too risky. Therefore, the development of reasonable chemoradiotherapy regimens and cycles is crucial in such cases to improve prognosis and enhance the 5-year survival rate. In the present case, radical surgery was chosen as the initial treatment option. Unfortunately, four months later, the patient developed bladder tumors and metastases. Depending on the overall condition of the patient and the specific pathology of the bladder, an individualized treatment plan involving chemotherapy and molecular targeted therapy may be considered.

The simultaneous occurrence of primary malignant neoplasms in the renal, ureteral, and urinary bladder regions poses diagnostic and therapeutic challenges. Distinguishing synchronous primaries from metastatic disease is crucial for guiding appropriate treatment strategies and prognostication. In this case, thorough clinical evaluation, imaging studies, and histopathological analysis facilitated accurate diagnosis and informed multidisciplinary management. The successful outcome underscores the importance of a comprehensive approach involving urological, oncological, and radiological expertise in addressing complex oncological presentations. Furthermore, this case highlights the need for heightened awareness of synchronous primary malignancies in clinical practice, particularly in regions with a high incidence of genitourinary malignancies such as China. In addition, multidisciplinary treatment and individualized precision treatment strategies may help improve the prognosis of MPMNs (19).The exceptional nature of this case underscores the imperative for systematic data collection on analogous instances in future investigations, coupled with comprehensive research into molecular mechanisms to elucidate the underlying pathophysiological determinants of such rare co-occurrences. Regrettably, we were unable to acquire the freshly procured clinical specimens from the patient, thereby precluding the implementation of genetic screening. Had the genetic screening been feasible, it would have facilitated a comprehensive elucidation of potential genetic predisposition and somatic mutation-driven molecular pathways associated with the disease. This would have enabled the provision of personalized genetic counseling and early cancer risk assessment for the patient’s familial cohort.

## Conclusions

This case report illustrates a rare occurrence of synchronous primary malignant neoplasms affecting the renal, ureteral, and urinary bladder regions in a Chinese male patient. Through detailed clinical description and analysis, we emphasize the importance of comprehensive evaluation and multidisciplinary management in addressing complex oncological presentations. Heightened awareness of synchronous primary malignancies is essential for guiding optimal treatment strategies and improving patient outcomes. Further research is warranted to elucidate the underlying mechanisms and risk factors associated with the development of synchronous primary malignancies in the genitourinary tract.

## Data Availability

The raw data supporting the conclusions of this article will be made available by the authors, without undue reservation.
